# Supplementary material for: Defining cell type-specific immune responses in a mouse model of allergic contact dermatitis by single-cell transcriptomics
Source: eLife. 2024 Aug 30;13:RP94698. doi: 10.7554/eLife.94698 (PMC11364439; doi:10.7554/eLife.94698)
Supplement: Supplementary file 1. [file elife-94698-supp1.docx]

**Table S1. List of gene primers used for RT-qPCR:**

| **Gene** | **Strand** | **Primer sequence** |
| --- | --- | --- |
| ***Tbp*** | Forward | CCTTGTACCCTTCACCAATGAC |
|  | Reverse | ACAGCCAAGATTCACGGTAGA |
| ***Col1a1*** | Forward | GCTCCTCTTAGGGGCCACT |
|  | Reverse | ATTGGGGACCCTTAGGCCAT |
| ***Il1b*** | Forward | GAAATGCCACCTTTTGACAGTG |
|  | Reverse | TGGATGCTCTCATCAGGACAG |
| ***Ly6g*** | Forward | GACTTCCTGCAACACAACTACC |
|  | Reverse | ACAGCATTACCAGTGATCTCAGT |
| ***Ifng*** | Forward | GCCACGGCACAGTCATTGA |
|  | Reverse | TGCTGATGGCCTGATTGTCTT |
| ***Il4*** | Forward | GAGCCATATCCACGGATGCGAC |
|  | Reverse | ATGCGAAGCACCTTGGAAGCCC |
| ***Il17a*** | Forward | ACGCGCAAACATGAGTCCAGGG |
|  | Reverse | TGAGGGATGATCGCTGCTGCCT |
| ***Mcpt8*** | Forward | AACGCTGAAGGAGGGGAAATCA |
|  | Reverse | TTGCCACCAGGAAACCACCA |
| ***Cma1*** | Forward | CACGGAGTGCATACCACACT |
|  | Reverse | AAGCTTCTGCCACGTGTCTT |
| ***Cd68*** | Forward | CTTCCCACAGGCAGCACAG |
|  | Reverse | AATGATGAGAGGCAGCAAGAGG |
| ***Cxcl9*** | Forward | GGAGTTCGAGGAACCCTAGTG |
|  | Reverse | GGGATTTGTAGTGGATCGTGC |
| ***Cxcl10*** | Forward | CCACGTGTTGAGATCATTGCCACG |
|  | Reverse | ATCCATCGCAGCACCGGGGT |
| ***Cxcr3*** | Forward | TACCTTGAGGTTAGTGAACGTCA |
|  | Reverse | CGCTCTCGTTTTCCCCATAATC |
| ***Cd8a*** | Forward | GACCGGATTGGACTTCGCCT |
|  | Reverse | GACTAGCGGCCTGGGACATT |
| ***Il10*** | Forward | GGCGCTGTCATCGATTTCTCCCC |
|  | Reverse | GGCCTTGTAGACACCTTGGTCTTGG |
| ***Il13*** | Forward | TGCTTGCCTTGGTGGTCTCGC |
|  | Reverse | GCGGCCAGGTCCACACTCCA |
| ***Cd4*** | Forward | CTAGCTGTCACTCAAGGGAAGA |
|  | Reverse | CGAAGGCGAACCTCCTCTAA |
| ***Ccl2*** | Forward | CACAGTTGCCGGCTGGAGCA |
|  | Reverse | CAGCAGGTGAGTGGGGCGTT |
| ***Stat1*** | Forward | TCACAGTGGTTCGAGCTTCAG |
|  | Reverse | CGAGACATCATAGGCAGCGTG |
| ***Apoe*** | Forward | CTGACAGGATGCCTAGCCG |
|  | Reverse | CGCAGGTAATCCCAGAAGC |
| ***Ifngr1*** | Forward | TGACTATGCACGGTCAAAAGAG |
|  | Reverse | ATTCACAACGACTTCAGGGTG |
